# Suicide Attempt by Cement Ingestion: A Case Report

**DOI:** 10.7759/cureus.31650

**Published:** 2022-11-18

**Authors:** Hanan Aly, Ammar Y Bafarat, Alaa M Alzamil, Inas S Bagabas, Suhail A Labban

**Affiliations:** 1 Psychiatry and Behavioral Sciences, King Abdulaziz Hospital, Jeddah, SAU; 2 Psychiatry and Behavioral Sciences, Sohag University, Sohag, EGY; 3 General Practice, King Abdulaziz Hospital, Jeddah, SAU; 4 General Practice, King Saud Bin Abdulaziz University for Health Sciences College of Medicine, Riyadh, SAU; 5 Medicine, King Saud Bin Abdulaziz University for Health Sciences College of Medicine, Jeddah, SAU

**Keywords:** suicide attempt, foreign body ingestion, suicide, cement ingestion, cement

## Abstract

A 30-year-old Pakistani construction worker, not known to have any chronic medical illnesses, presented to the emergency room with a history of ingesting two cups of cement diluted in water, seven hours prior to the presentation, in addition to a cut on his left wrist using a sharp piece of ceramic. He was conscious, oriented, and vitally stable. Physical examination was unremarkable except for epigastric hardness and tenderness. Treatment upon admission included escitalopram 10 mg and haloperidol 5 mg. Upper GI endoscopy showed large, hard cement in the stomach and multiple pre-antral erosions. The patient was started on omeprazole 40 mg after the procedure. Exploratory laparotomy and gastrotomy were performed as well. The procedure showed a foreign body, gypsum, occupying the stomach and extending from the fundus to the pylorus. Multiple small foreign bodies were seen in the rectum. The foreign bodies were extracted completely. Before discharge, a suicide risk assessment was done using the modified SAD PERSONS scale. The patient’s total score was 5, which is low risk. The patient received psychiatric care, and his post-discharge follow-up was unremarkable.

## Introduction

Suicide accounts for one out of every 100 fatalities worldwide, making it one of the main causes of death [1]. According to the World Health Organization (WHO), over 77% of suicide deaths worldwide occur in low and middle-income countries (LMICs). Globally, suicide is the second leading cause of premature mortality in individuals aged 15 to 29 years [2]. Suicide is thus considered a global public health concern [1].

A family history of suicide, genetic predisposition, psychosocial pressures, underlying mental disorders, traumatic life events, personality traits, and cognitive distortions are only a few of the risk factors that might lead to suicide [3]. Research about suicide has increasingly concentrated on biological suicide indicators as early identification of suicidal behaviors can help with suicide prevention and therapy [4].

A retrospective study conducted in the eastern region of Saudi Arabia reported that the highest percentage of suicide was among non-Saudis (83%). The second highest percentage was among Saudi nationals (15%). Suicide by hanging was the most common method among all cases, followed by firearm death, falling from a height, and poisoning. Suicide attempts by cement ingestion are very unusual, both in the Kingdom of Saudi Arabia and the world. Therefore, a standard of care for such attempts has not been established. In this report, we present a successful treatment of cement ingestion by a surgical gastrotomy [5].

## Case presentation

A 30-year-old male, Pakistani construction worker, not known to have any chronic medical illnesses, presented to the emergency room with a history of ingesting two cups of cement diluted in water, seven hours prior to the presentation. He also presented with a cut on his left wrist using a sharp piece of ceramic. He had dull epigastric pain and nausea. He tried to vomit the material but failed. He was admitted for exploratory laparotomy, gastrotomy, and foreign body extraction.

Taking history from the patient was challenging due to a language barrier, as he only spoke Urdu. With the assistance of a translator, the patient reported that he ingested the material in an attempt of suicide following an argument with his family over the phone. He had no intention or plan of ending his life prior to the family issue. He denied any previous attempts. He also denied any history of psychiatric disorders or being on any psychiatric medications. He denied substance use. The psychiatric evaluation revealed that he had no symptoms or signs suggestive of depression. He felt guilty about the attempt and said that he had no intention of committing suicide again as god had given him a new life.

Physical examination was unremarkable except for epigastric hardness and tenderness. The patient was conscious, oriented, and vitally stable. He was started on escitalopram 10 mg tablet orally (PO) once a day (OD) and haloperidol 5 mg pro re nata (PRN) IV/intramuscular (IM) despite not fulfilling any mood disorder criteria in the Diagnostic and Statistical Manual of Mental Disorders, 5th edition (DSM-5), as the attempt was due to a stressful event. Four-point restraint was recommended in case of agitation, aggression, or suicide attempt.

**Figure 1:**
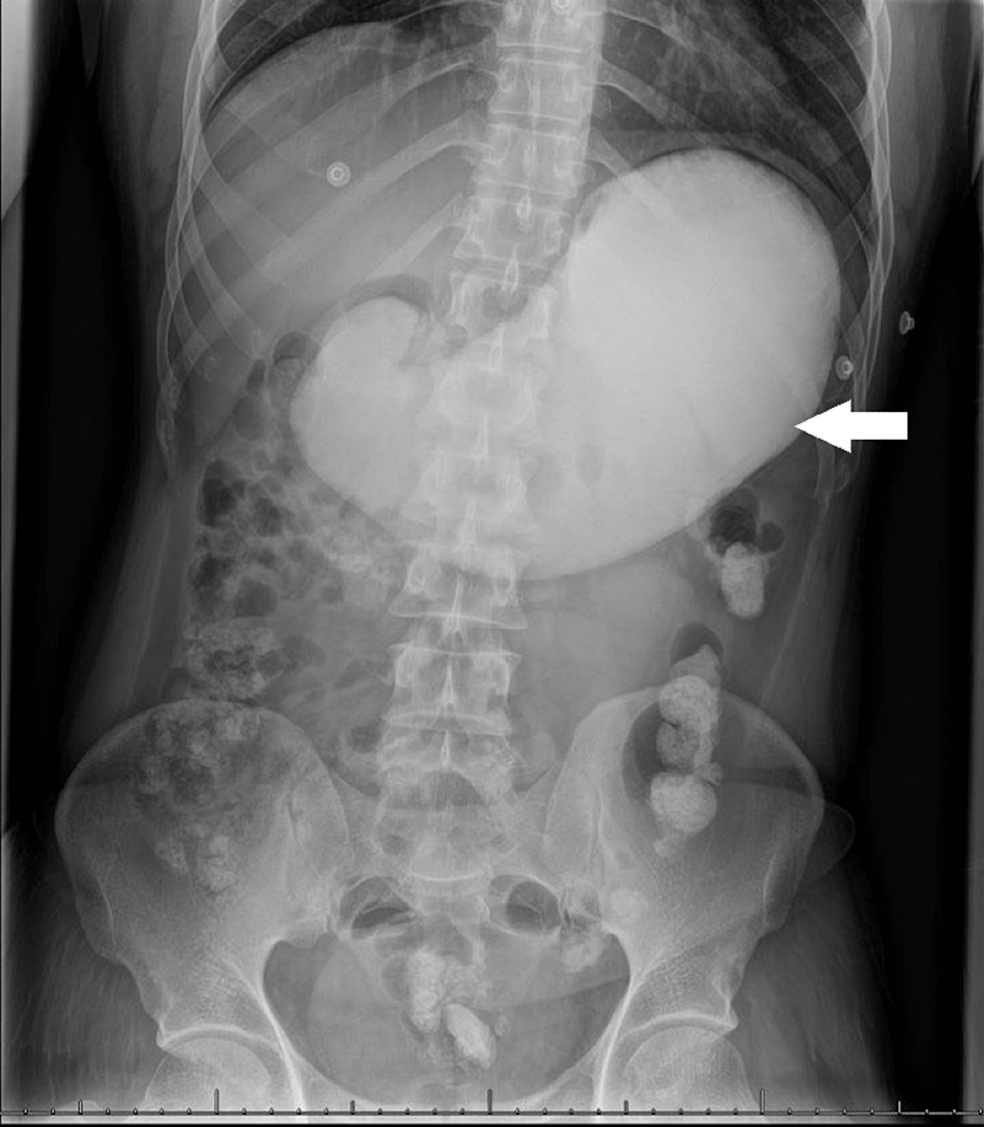
Abdominal X-ray Radiograph of the abdomen (first hospitalization day) showing dilated stomach with radiopaque shadow.

Several investigations were conducted. Abdominal X-ray showed dilated stomach with a radiopaque shadow (Figure 1). Upper GI endoscopy showed large, hard cement in the stomach that took the shape of the stomach body. It also showed multiple pre-antral erosions. The esophagus was normal. No ulcerations or materials were seen in it. Duodenum was not examined. The patient was started on omeprazole 40 mg IV OD after the procedure.

**Figure 2:**
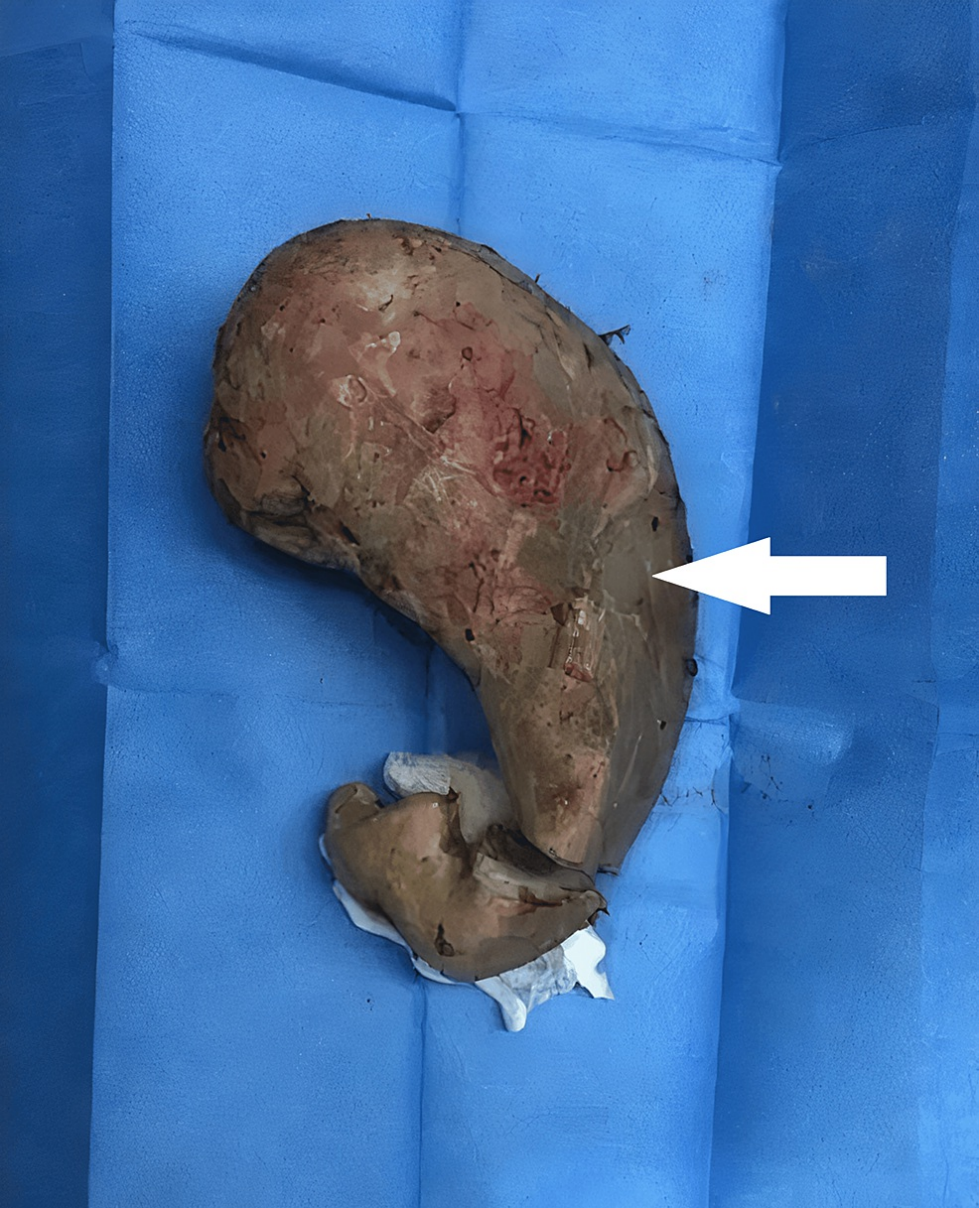
Extracted cement

Exploratory laparotomy and gastrotomy were performed as well. An upper midline 10 cm incision away from the pylorus and gastroesophageal junction was made. The procedure showed a foreign body, gypsum, occupying the stomach and extending from the fundus to the pylorus. Multiple small foreign bodies were seen in the rectum. The foreign bodies were extracted completely (Figure 2). No foreign bodies or perforations were seen in the small bowels.

Pulmonary CT angiography showed mild left pleural effusion and bilateral basal patchy consolidation. It was negative for pulmonary embolism. Abdominal and pelvic CT with IV and oral contrast was also done to rule out the intra-abdominal collection.

On the third day post exploratory laparotomy and gastrotomy, clinical examination and history revealed minimal abdominal pain and purulent and yellowish discharge at the proximal part of the wound site. It was irrigated with normal saline (NS). The patient was started on ciprofloxacin 500 mg tablet PO/twice a day (BID) and metronidazole 500 mg tablet PO/three times a day (TID). A wound culture was ordered. Three days later, the result came back positive for *Pseudomonas aeruginosa* and *Enterobacter cloacae*, for which he was switched on to meropenem 500 mg IV TID. The patient passed flatus but did not pass stool. He had no nausea or vomiting. He was also found to have hypokalemia and hypomagnesemia, which was corrected. Moreover, on the eighth day postoperatively, he underwent debridement and fascial closure, as the exploratory laparotomy and gastrotomy were complicated by fascial dehiscence.

Before discharge, a suicide risk assessment was done using the modified SAD PERSONS scale [6]. The patient’s total score was 5, which is low risk. The patient received psychiatric care, and his post-discharge follow-up was unremarkable.

## Discussion

Cement is an easily accessible substance. When it is diluted in water, the base of tri-calcium silicate rapidly reacts to release calcium ions and hydroxide ions causing an increase in pH and a large amount of heat.

It is well known that skin contact with wet cement can severely damage the skin and cause burns that have an insidious onset and a progressive nature. There are multiple reports in the literature on these topical burns that are induced by contact with wet cement. However, suicide attempts by cement ingestion are extremely rare. Therefore, a clear guideline for treatment has not been established yet [7].

Previous reports showed different approaches to treating the patients due to the variety in the amount of cement ingested by each patient, time of presentation, and complications.

In one case, a 7-cm hyperdense material noted on the CT scan was removed by endoscopy only, and the patient made full recovery [8].

Another case series published in 2005 included six cases, two of which had to eventually undergo a gastrotomy to remove the material. The remaining four patients did not have an opacity on their radiographs. Thus, surgery was not needed. Gastric lavage with normal saline was used in three out of the six cases to prevent solidification. It was of no benefit as the cement hardens after three hours, and gastric lavage is not indicated after solidification [9].

The latest case report in 2017 recommended early endoscopy to rule out caustic injury to the esophagus and stomach. They avoided suctioning the material. They also avoided gastric lavage to prevent any further injuries as a result of the reaction between water and cement. The patient was treated conservatively, and no surgery was needed [10].

## Conclusions

The ingestion of cement is a high-risk situation that might lead to serious complications and require immediate surgical intervention. We suggest checking the status of the upper GI tract and removing the cement as soon as possible. Once the stabilization of the patient is accomplished and their safety is ensured, a suicide risk assessment and a comprehensive psychiatric evaluation must be performed.
